# Risk factors associated with obstructive sleep apnea-hypopnea syndrome in Chinese children: A single center retrospective case-control study

**DOI:** 10.1371/journal.pone.0203695

**Published:** 2018-09-13

**Authors:** Ling Shen, Zongtong Lin, Xing Lin, Zhongjie Yang

**Affiliations:** Department of Otolaryngology, Fuzhou Children’s Hospital of Fujian Province, Fujian Medical University Hospital, Fuzhou, China; Ospedale S. Corona, ITALY

## Abstract

Pediatric obstructive sleep apnea-hypopnea syndrome is caused by multiple factors. The present study aimed to investigate the potential risks of pediatric obstructive sleep apnea hypopnea syndrome (OSAHS) and their correlation with the disease severity. A total of 338 pediatric patients with OSAHS (polysomnography (PSG) diagnosis) were enrolled between June 2008 and October 2010. These pediatric patients were divided into mild, moderate and severe subgroups according to the obstructive apnea index (OAI) and/or apnea hypoventilation index (AHI). A total of 338 pediatric patients with vocal nodules who were without obstruction of the upper respiratory tract were enrolled as the control group. The patients were analyzed retrospectively. The average number of upper respiratory tract infections each year and tonsil hypertrophy, adenoid hypertrophy, positive serum tIgE, chronic sinusitis, nasal stenosis, craniofacial features and obesity were significantly higher in OSAHS compared with controls (*P*<0.01). The parameters the average number of upper respiratory tract infections each year (OR: 1.395, 95% CI: 1.256–1.550), adenoid hypertrophy (OR: 8.632, 95% CI: 3.990–18.672), tonsil hypertrophy (OR: 9.138, 95% CI: 4.621–18.073), nasal stenosis (8.023, 95% CI: 3.633–17.717) and chronic sinusitis (OR: 27.186, 95% CI: 13.310–55.527) were independent factors of pediatric OSAHS (*P*<0.01). The distribution of chronic sinusitis, nasal stenosis, craniofacial features and obesity indicated a gradual increasing trend in the severity of OSAHS (*P*<0.01). Number of upper respiratory tract infections per year, adenoid hypertrophy, tonsil hypertrophy, chronic sinusitis, nasal stenosis, infections, allergic reactions, craniofacial features and obesity may be potential risk factors of pediatric OSAHS.

## Introduction

Pediatric obstructive sleep apnea-hypopnea syndrome (OSAHS) is a breathing disorder characterized by recurrent complete and/or partial upper airway obstruction during sleep; it interrupts normal ventilation in sleep resulting in intermittent hypoxia and hypercapnia, frequent arousals, and sleep fragmentation [1]. It may result in symptoms of habitual snoring (commonly with intermittent pauses, snorts, or gasps), disturbed sleep pattern, and daytime neurobehavioral problems [1]. This disturbs the normal ventilation and sleep of children, thus leading to a series of pathophysiological changes, such as growth restriction, behavioral abnormalities and cardiovascular disease. OSAHS is a common disease in children with the incidence rate of 1–5% [1–3]. It rarely leads to death [4].

The majority of the studies have concluded that the main causes of pediatric OSAHS are adenoid hypertrophy and tonsil hypertrophy and that adenotonsillectomy is the main treatment [1, 4–7] with a 90% maximum efficiency [8]. Nevertheless, certain studies have suggested that adenoid hypertrophy and tonsil hypertrophy in children are not significantly correlated with pediatric OSAHS [9]. Moreover, the clinical symptoms do not improve significantly following resection of hypertrophic adenoids and tonsils, and recurrence occurs after improvement, suggesting that there may be other causes beside adenoid hypertrophy and tonsil hypertrophy that are responsible for the development of pediatric OSAHS. Furthermore, it has been reported that pediatric chronic sinusitis is closely related to adenoids [10] and infections of the oral cavity and upper respiratory tract may also play a role [11–13]. It is reasonable to speculate that nasal diseases may play significant roles in pediatric obstructive sleep apnea hypopnea syndrome. In addition to the aforementioned factors that directly affect ventilation of the upper respiratory tract, the latter process is further affected by systemic factors such as obesity and hypothyroidism. Maternal smoking has been shown to lead to disturbances of the children’s sleeping pattern [14] and a cigarette smoke environment has been associated with OSAHS [15, 16]. Therefore, pediatric OSAHS can be caused by multiple factors.

Nevertheless, several different risk factors have been reported for the development of pediatric OSAHS. Redline et al. reported that the incidence rate of OSAHS in African-American children is higher than that noted in other ethnic groups [17]. Thus, it can be inferred that the genetic diversity of ethnic groups can lead to different incidence of OSAHS, which is dependent on the potential risk factors reported by several studies. A limited number of studies with large sample size have investigated the risk factors of OSAHS in Chinese children. Consequently, the present study retrospectively analyzed the potential risk factors of OSAHS in Chinese children that were treated in the Otorhinolaryngology department of the Fuzhou children’s hospital of Fujian province, and investigated the relationship of these factors with the severity of the disease. The study further aimed to explore the therapeutic applications with regard to OSAHS.

## Materials and methods

### Study design and participants

Based on the diagnostic criteria of the “diagnosis and treatment guidelines of pediatric obstructive sleep apnea hypopnea syndrome (Urumqi)” in 2007 [18], the present study enrolled 338 pediatric patients with OSAHS who had complete clinical data and were treated in the outpatient department and wards of the Otorhinolaryngology department of the Fuzhou children’s hospital of Fujian province between June 2008 and October 2010. These patients were classified as the case group (OSAHS group). The screening criteria of the pediatric patients in the case group were as follows. Inclusion criteria: 1) patients who met Urumqi criteria of the pediatric OSAHS and were diagnosed by polysomnography (PSG); and 2) patients who were ≤14 years of age and had not reached puberty. The exclusion criteria were: 1) patients who had previously undergone tonsillectomy and adenoidectomy because it is a risk factor for recurrent snoring [1]; 2) patients who presented with growth and developmental disorders, which may be associated with poor breathing functions; 3) patients who experienced neuromuscular disease, which may also be associated with poor breathing functions; or 4) patients with previous severe diseases in vital organs such as heart, lung, liver, kidney or brain and/or history of major trauma, because the impact of those diseases on the development of OSAHS is unknown and it would introduce a bias.

During the same period, 338 pediatric patients (age and gender matched with those in the case group) with vocal nodules who had complete clinical data were enrolled as the control group. The main symptom of the pediatric patients in the control group was hoarseness, although they had no symptoms of upper airway obstruction such as nasal congestion, mouth breathing and snore. Moreover, the selection criteria for the control group included negative PSG results. The present study was approved by the Ethics Committee of the Fuzhou Children’s Hospital of Fujian province. The study was approved by the institutional review board of the Fuzhou Children’s Hospital of Fujian province (number: 2010–001). The data were anonymous, and the requirement for informed consent was therefore waived.

### Collection of clinical data

Sleep monitoring was conducted for all the enrolled pediatric patients in the case group. Mouth and nasal airflow, thoracoabdominal breathing movements, blood oxygen saturation, snoring index, EEG, EOG, mandibular electromyography and positions were monitored for at least 8 h of night sleep. According to the diagnostic criteria [18], the time of respiratory event was 5 s, and obstructive apnea was defined as the presence of thoracoabdominal movements, in the absence of mouth and nasal breathing for 5 s and/or longer (≥5 s). Low ventilation was defined as the signal amplitude of mouth and nasal breathing that was decreased by 50% and/or more (≥50%), with a duration higher than and/or equal to 5 s (≥5 s) that was accompanied by a decrease in blood oxygen saturation of higher than and/or equal to 3% (≥3%). A mild disease was defined by a frequency of 5 to 10 times/h of AHI, whereas a frequency of 10 to 20 times/h was considered as moderate disease and a frequency of 20 times/h and/or higher (>20 times/h) as severe disease. In addition, according to the diagnostic criteria of the upper respiratory tract infections [19], the retrospective disease history of the pediatric patients provided by their parents was collected. This information included symptoms such as nasal congestion, running nose, cough, fever and sore throat. Moreover, examinations and diagnostic records of pharyngeal hyperemia and tonsil enlargement were analyzed. The number of upper respiratory tract infections that were noted each year in the case and the control groups were recorded. For the purpose of this study, the assessors were not told of the diagnosis of the patients they assessed and were therefore blind to grouping.

**Fig 1:**
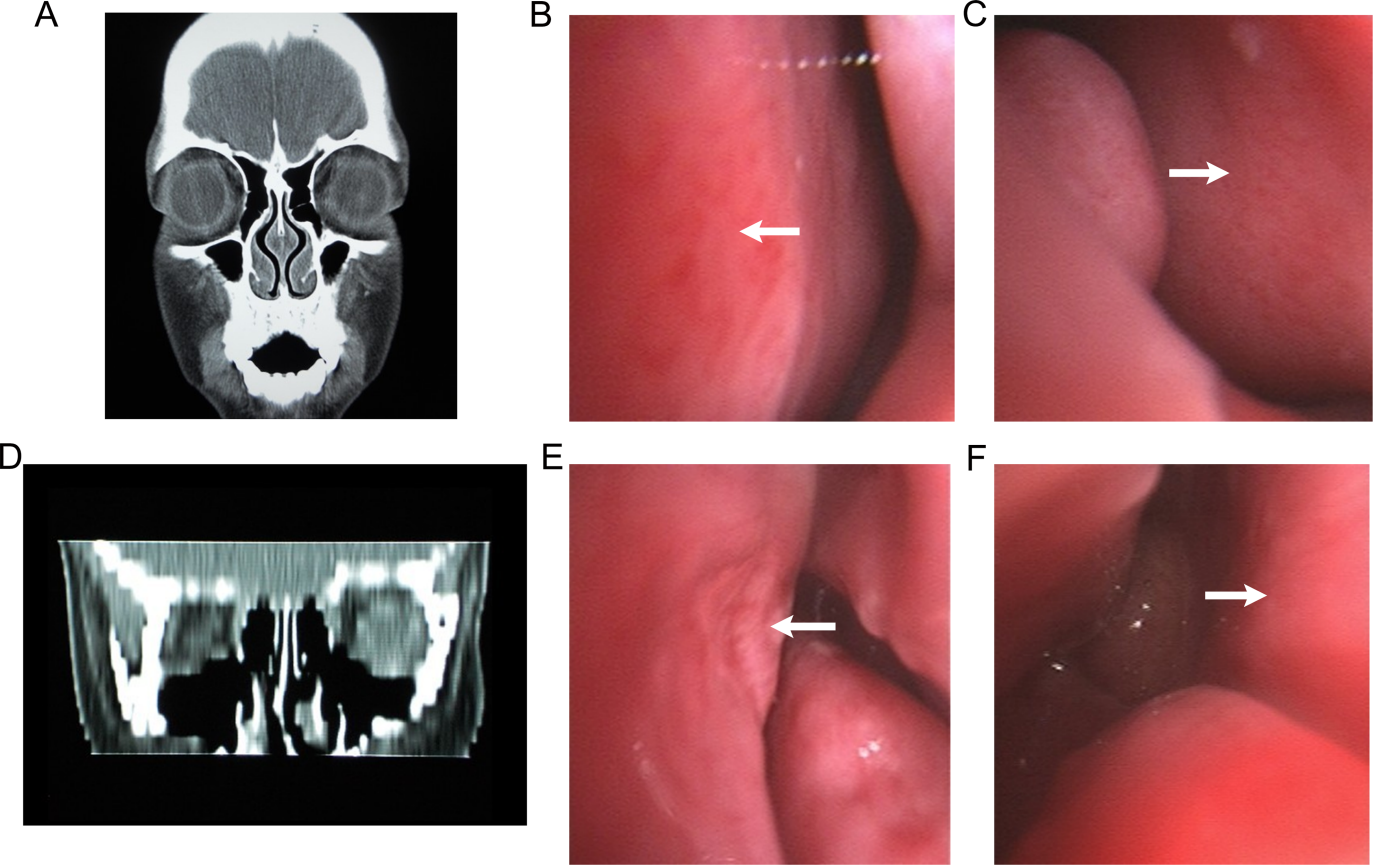
Results of paranasal sinus CT and electron-nasopharyngolaryngoscopy for typical cases with nasal stenosis. Electron-nasopharyngolaryngoscopy indicated the presence of bilateral nasal mucosal hyperemia and swelling, middle and inferior turbinate hypertrophy, as well as aperture-like stenosis of the bilateral nasal cavities.

The pharynxes of the pediatric patients in the case and the control groups were examined by the method of mouth opening and tongue pressing. The tonsil size was divided into grades I to IV [20, 21]. Grade I was confined by tonsil fossa. Grade II was protruding from the palatoglossal arch, occupying half of the pharyngeal space. Grade III was protruding from the tonsil fossa, occupying 75% of the pharyngeal space. Grade IV blocked the pharyngeal space since the bilateral tonsils were almost folded. Tonsil hypertrophy was defined as grades III to IV of tonsils and accompanied by clinical symptoms [22]. All the pediatric patients were examined by electron-nasopharyngolaryngoscopy (PENTAX VNL-1530T). The pediatric patients were in supine position, and the examination was initiated following convergence of nasal mucosa. Local anesthesia was conducted using 0.5% and 1% of ephedrine that were placed in the nasal cavity alternately (three drops for each nostril at a rate of 1 drop/min). The electron-nasopharyngolaryngoscope was inserted into the nasal cavity from one of the anterior nares, reaching the back of the nasal cavity through the wider middle meatus and/or inferior meatus. Subsequently, the posterior nostril and nasopharyngeal structures, such as adenoids that were exposed to the posterior nostril were visible. Following this process, the electron-nasopharyngolaryngoscope was further inserted to the nasopharynx from the posterior nostril until the throat. The other side was examined using the same method. The following features were carefully observed: the color of nasal mucosa and turbinate, the swelling condition, the location and amount of secretions, the presence of nasal septum ectasia and/or nasal septum deviation, the presence and the degree of nasal stenosis, the relationship between adenoids and posterior nostrils, tonsil size, surface conditions and the degree of pharyngeal stenosis. The diagnosis was defined as rhinitis combined with history of nasal congestion in case of nasal mucosa and turbinate swelling, pale or hyperemia, and in the presence of accumulation of nasal secretions. Sticky purulent secretions from the middle meatus and/or mucosal edema and/or nasal polyps of the middle meatus were diagnosed as chronic sinusitis [23]. The gap of the narrowed nasal cavity was diagnosed as nasal stenosis (diagnostic gold standard) by electron-nasopharyngolaryngoscopy. The results of sinus CT and video laryngoscopy of typical nasal stenosis were shown in Fig 1. The obstruction of the posterior nostrils by adenoid enlargement lower than and/or equal to 25% (≤25%) was classified as grade I, whereas at a percentage of 26 to 50%, 51 to 75% and 76 to 100% as grade II, III and IV, respectively. Adenoid hypertrophy was defined as Grades III to IV adenoids that were accompanied by clinical symptoms [24].

Serum tIgE was detected *in vitro* using the HITACHI MAST CLA multi-allergen detection system (MAST (CLA-1 type), HITACHI, Japan) for all pediatric patients in the case and the control groups. This is a cost effective, compact, and flexible system that measures a patient’s response to several allergens simultaneously with a single serum sample. It is therefore less invasive for the patient, which is of value in children. The results of the tIgE test were classified into six grades based on the diagnostic criteria recommended by the instructions of the detection system (Table 1). Positive serum tIgE indicated the presence of allergic reactions [25, 26] and upper airway diseases of the pediatric patients that correlated with allergic reactions. All biochemical tests were performed at the hospital’s central laboratory and the technicians were blind to grouping. The heights and weights of the pediatric patients in the case and the control groups were measured when they sought medical care. According to the “standardized growth curve of the heights and weights of Chinese children and adolescents aged 0–18 years” in 2009 [27], the patients with weights of more than 20% of the mean value of the weight of their peers of the same gender were diagnosed as obese, whereas patients with weights that were higher than the range between 20% and 29% of the mean value were diagnosed as mildly obese. The patients with weights higher than the range of 30% to 49% of the mean value were diagnosed as moderate obese, and patients with weights of more than and/or equal to 50% of the mean value were diagnosed as severely obese [19]. At present, a few studies have examined the craniofacial features of pediatric OSAHS. Consequently, a lack of quantitative diagnostic criteria exists [28–30]. In the present study, the judgment was based on the typical appearances such as long narrow face, small jaw, mandibular retrusion, cross-bite and flat bridge of the nose.

**Table 1:** Judgment criteria of serum tIgE.

Grading*	tIgE concentration (IU/ml)	Judgment
4	>10.00	Very high
3	>5.00	high
2	>2.50	Medium high
1	>0.70	Medium low
1/0	>0.22	Very low
0	0	Cannot be detected

Note

* tIgE was positive if it was ≥1, and negative if it was ≤1/0.

### Statistical analysis

The data were encoded and the authors that performed the statistical analysis were therefore also blind to grouping. The normal distribution of the continuous data was tested by the Kolmogorov-Smirnov test. Continuous data that followed normal distribution were represented by means ± SD, whereas in any other case the median (range) was used. The continuous data between the two groups that followed normal distribution were represented by the student *t* test. The categorical data and/or grade data were expressed as n (%). Statistical analysis was carried out by the Chi-square and/or the rank sum tests. Moreover, univariable and multivariable logistic regression analyses were conducted. The parameters that were significantly different as determined by univariable analyses were included in the multivariable logistic regression analysis. The process of parameter inclusion was set to forward (the dependent parameter y was the presence of OSAHS, and the independent parameters were various potential risk factors). Correlation analysis was conducted by Pearson and/or Spearman correlation analysis. SPSS 17.0 software package was used (IBM, Armonk, NY, USA). A P value of lower than 0.05 (*P*<0.05) was considered statistically significant.

## Results

### Baseline characteristics of the pediatric patients in the case and control groups

A total of 338 pediatric patients were selected in the case group, including 270 boys and 68 girls, with an average age of 5.80 ± 2.45 years. The control group included 338 pediatric patients, consisting of 270 boys and 68 girls, with an average age of 5.79±2.48 years. The age and gender were matched between the two groups. The proportion of age distribution in the case group indicated that it gradually increased starting from 3 years old, reached the peak at 6 years old and then gradually decreased. The number of upper respiratory tract infections per year and the incidence of adenoid hypertrophy, tonsil hypertrophy, chronic sinusitis, nasal stenosis, positive serum tIgE, craniofacial features and obesity in the case group was significantly higher compared with that noted in the control group (*P*<0.001). The aforementioned parameters were potential risk factors for the development of pediatric OSAHS, but the incidence of rhinitis was not statistically significant between the two groups (*P* = 0.132) (Table 2).

**Table 2:** Comparison of baseline data of the pediatric patients between the case and the control groups.

Parameters		Control group (n = 338)	Case group (n = 338)	P value
Gender	Male	270 (79.9%)	270 (79.9%)	1
	Female	68 (20.1%)	68 (20.1%)	
Age		5.79±2.48	5.80±2.45	0.964
Obesity	No	288 (85.2%)	253 (74.8%)	0.001
	Yes	50 (14.8%)	85 (25.2%)	
Average number of upper respiratory infection/year		4.49 ± 2.57	8.72±5.59	<0.001
Adenoid hypertrophy	No	235 (69.5%)	35 (10.3%)	<0.001
	Yes	103 (30.5%)	303 (89.7%)	
Tonsil hypertrophy	No	294 (87.0%)	107 (31.6%)	<0.001
	Yes	44 (13.0%)	231 (68.4%)	
Chronic sinusitis	No	298 (88.2%)	68 (20.1%)	<0.001
	Yes	40 (11.8%)	270 (79.9%)	
Nasal stenosis	No	317 (93.8%)	169 (50.0%)	<0.001
	Yes	21 (6.2%)	169 (50.0%)	
Positive serum tIgE	No	229 (67.8%)	132 (39.1%)	<0.001
	Yes	109 (32.2%)	206 (60.9%)	
Craniofacial features	No	331 (97.9%)	299 (88.5%)	<0.001
	Yes	7 (2.1%)	39 (11.5%)	
Rhinitis	No	285 (84.3%)	270 (79.9%)	0.132
	Yes	53 (15.7%)	68 (20.1%)	

Note: Adenoid hypertrophy was defined as grade III to IV adenoids, while tonsil hypertrophy was defined as grade III to IV tonsils.

### Evaluation of the risk factors of OSAHS by multivariable logistic regression analysis

The parameters that were significantly different as determined by univariable analyses were included in the logistic regression analysis, and the results showed that independent risk factors of pediatric OSAHS included adenoid hypertrophy, tonsil hypertrophy, nasal stenosis, chronic sinusitis and the average number of upper respiratory tract infection/year (Table 3). Nevertheless, positive serum total IgE, craniofacial features and obesity were not independent risk factors of pediatric OSAHS (Table 3). In addition, according to the OR value, the incidence of pediatric OSAHS with chronic sinusitis was 27.186 times higher than that noted in the absence of chronic sinusitis (95%CI: 13.310–55.527, *P*<0.001), whereas the incidence of nasal stenosis was 8.023 times higher than that noted in the absence of nasal stenosis (95%CI: 3.633–17.717, *P*<0.001). The incidence of tonsil hypertrophy was 9.138 times higher than that noted in the absence of tonsil hypertrophy (95%CI: 4.621–18.073, *P*<0.001), while the incidence of adenoid hypertrophy was 8.632 times higher than that noted in the absence of adenoid hypertrophy (95%CI: 3.990–18.672, *P*<0.001). With an increase in the number of upper respiratory tract infection, OR was increased by 1.395 times (95%CI: 1.256–1.550, P<0.001). (Table 3).

**Table 3:** Multivariable logistic regression analysis of the influencing factors of pediatric OSAHS.

	P value	OR value	95% CI	
			Lower limit	Upper limit
Adenoid hypertrophy	<0.001	8.632	3.990	18.672
Tonsil hypertrophy	<0.001	9.138	4.621	18.073
Chronic sinusitis	<0.001	27.186	13.310	55.527
Nasal stenosis	<0.001	8.023	3.633	17.717
Average number of upper respiratory tract infections/year	<0.001	1.395	1.256	1.550
Positive serum tIgE	0.177	1.537	0.824	2.868
Craniofacial features	0.198	2.335	0.643	8.482
Obesity	0.335	1.539	0.640	3.698

### Stratification analysis of the risk factors and disease severity

The correlation of different sizes of adenoids and tonsils with pediatric OSAHS in the case and control groups was further analyzed, and the results demonstrated that the OR value of pediatric OSAHS with grade III adenoids was 0.020 (95% CI: 0.010–0.039) compared with grade IV adenoids. In contrast to grade IV adenoids, the OR value for grade II and grade I adenoids was 0.006 (95% CI: 0.003–0.012) and 0.015 (95% CI: 0.007–0.033), respectively. The OR value of pediatric OSAHS with grade III tonsils was 0.182 (95% CI: 0.081–0.409) compared with grade IV tonsils. In contrast to grade IV tonsils, the OR values for grade II and grade I tonsils were 0.053 (95% CI: 0.025–0.115) and 0.008 (95% CI: 0.003–0.018), respectively. The chi-square test revealed that the grading of adenoids and tonsils in the case group gradually increased (*P* for trend <0.01), suggesting that the larger the adenoids and tonsils, the more prone were the subjects to the development of pediatric OSAHS (Table 4).

**Table 4:** Tonsil and adenoid sizes of children in the case and the control groups.

Parameters		Control group (n = 338)	OSAHS group (n = 338)	P value	OR value (95%CI)
Adenoid grading	Grade IV	14 (4.2%)	269 (79.6%)		1.00
	Grade III	89 (26.3%)	34 (10.1%)	<0.001	0.020(0.010, 0.039)
	Grade II	183 (54.1%)	20 (5.9%)	<0.001	0.006 (0.003, 0.012)
	Grade I	52 (15.4%)	15 (4.4%)	<0.001	0.015(0.007, 0.033)
Tonsil grading	Grade IV	8 (2.4%)	127 (37.6%)		1.00
	Grade III	36 (10.6%)	104 (30.8%)	<0.001	0.182 (0.081, 0.409)
	Grade II	98 (29.0%)	83 (24.5%)	<0.001	0.053 (0.025, 0.115)
	Grade I	196 (58.0%)	24 (7.1%)	<0.001	0.008 (0.003, 0.018)

### Correlation analysis of the risk factors with the severity of pediatric OSAHS

In the present study, pediatric patients in the case group were further divided into three sub-groups including mild disease (n = 176), moderate disease (n = 92) and severe disease (n = 70) according to the diagnostic criteria of the “diagnosis and treatment guidelines of pediatric obstructive sleep apnea hypopnea syndrome (Urumqi)” in 2007. The results of the rank correlation analysis demonstrated that adenoid hypertrophy and tonsil hypertrophy in the mild, medium and severe subgroups gradually increased (correlation coefficient r>0, *P*<0.01), suggesting that the larger the adenoids and tonsils, the more severe the development of the pediatric OSAHS (Table 5).

**Table 5:** The correlation of different sizes of adenoids and tonsils with the severity of OSAHS in the pediatric patients with OSAHS in the case group.

		Mild in OSAHS (n = 176)	Medium in OSAHS (n = 92)	Severe in OSAHS (n = 70)	Pearson correlation	r value of Spearman correlation coefficient	P value
Adenoid grading	Grade I	12 (6.8%)	3 (3.3%)	0 (0%)	0.227	0.214	<0.001
	Grade II	17 (9.7%)	3 (3.3%)	0 (0%)			
	Grade III	19 (10.8%)	11 (12.0%)	4 (5.7%)			
	Grade IV	128 (72.7)	75 (81.5%)	66 (94.3%)			
Tonsil grading	Grade I	18 (10.2%)	4 (4.3%)	2 (2.9%)	0.187	0.178	<0.001
	Grade II	49 (27.8%)	23 (25%)	11 (15.7%)			
	Grade III	52 (29.5%)	31 (33.7%)	21 (30%)			
	Grade IV	57 (32.4)	34 (37.0%)	36 (51.4%)			

The rank sum test was conducted for factors such as chronic sinusitis, nasal stenosis, craniofacial features and obesity of the pediatric patients with OSAHS in the mild, moderate and severe subgroups. Among them, there were significant differences with regard to the parameters chronic sinusitis, nasal stenosis, craniofacial features and obesity (*P*<0.05), while there was no statistical significance noted with regard to the parameter positive serum total IgE (*P* = 0.372) (Table 6).

**Table 6:** Rank-sum test results of the influencing factors of OSAHS severity in the case group.

		Mild in OSAHS group (n = 176)	Medium in OSAHS group (n = 92)	Severe in OSAHS group (n = 70)	P value
Chronic sinusitis	Yes	124 (70.5%)	80 (87.0%)	66 (94.3%)	<0.001
	No	52 (29.5%)	12 (13.0%)	4 (5.7%)	
Nasal stenosis	Yes	74 (42.0%)	45 (49.0%)	50 (71.4%)	<0.001
	No	102 (58.0%)	47 (51.0%)	20 (28.6%)	
Positive serum tIgE	Yes	113 (64.2%)	51 (55%)	42 (60%)	0.372
	No	63 (35.8%)	41 (44%)	28 (40%)	
Craniofacial features	Yes	7 (4.0%)	13 (14.1%)	19 (27.1%)	<0.001
	No	169 (96%)	79 (85.9%)	51 (72.9%)	
Obesity	Yes	31 (17.6%)	26 (28.3%)	28 (40%)	<0.001
	No	145 (82.4%)	66 (71.7%)	42 (60%)	

A linear trend analysis was conducted for chronic sinusitis, nasal stenosis, craniofacial features and obesity, and the results indicated that the distribution of chronic sinusitis (Z = 2.046, *P* <0.05), nasal stenosis (Z = 2.812, *P*<0.05), craniofacial features (Z = 4.890, *P*<0.05) and obesity (Z = 3.234, *P*<0.05) gradually increased compared with the increase of the severity of OSAHS.

The pediatric patients in the case group were divided into the obesity and non-obesity groups based on whether the pediatric patients were obese. Fisher’s exact test demonstrated that the grading of adenoid and tonsil sizes was not significantly different between the obesity and non-obesity groups (*P*>0.05), although the parameters of the PSG of the obesity group were significantly elevated compared with those noted in the non-obesity group (*P*<0.001) (Table 7).

**Table 7:** Analysis of the severity of OSAHS in obese and non-obese children in the case group.

		Obesity pediatric patients (n = 85)	Non-obesity pediatric patients (n = 253)	P value
Adenoid grading	Grade I	3 (3.5%)	12 (4.7%)	0.578
	Grade II	4 (4.7%)	16 (6.3%)	
	Grade III	9 (10.6%)	25 (9.9%)	
	Grade IV	69 (81.2%)	200 (79.1%)	
Tonsil grading	Grade I	3 (3.5%)	21 (8.3%)	0.432
	Grade II	22 (25.9%)	61 (24.1%)	
	Grade III	27 (31.8%)	77 (30.4%)	
	Grade IV	33 (38.8%)	94 (37.2%)	
OSAHS severity	Mild	31 (36.5%)	145 (57.3%)	<0.001
	Moderate	26 (30.6%)	66 (26.1%)	
	Severe	28 (32.9%)	42 (16.6%)	

## Discussion

The findings of the present study suggest that OSAHS can occur at any age in childhood, although the incidence at each age group gradually increased starting from 3 years of age and peaking at 6 years; the incidence gradually decreased after 6 years of age. In addition to the bloom period of pediatric lymphoid tissue hyperplasia, the peak period of upper respiratory tract infection was estimated at the age group of 3 to 6 years. The study of Altken et al. demonstrated that this parameter was susceptible to cold during childhood with an average incidence number of 6 to 8 times/year [31]. In the present study, the average number of upper respiratory tract infection in each year in the case group was significantly higher compared with that noted in the control group, suggesting that pediatric OSAHS closely correlated with upper respiratory tract infections. In addition to infections, allergic inflammation of the upper respiratory tract has been recognized by a majority of studies as a risk factor of pediatric OSAHS. In the present study, a large number of pediatric patients with OSAHS combined with chronic sinusitis were noted. The results of the present study indicated that the proportion of positive serum tIgE in the case group was significantly higher compared with that noted in the control group. The allergens in the respiratory airflow can act on the nasal mucosa, adenoids and tonsils, inducing nasal inflammation, chronic sinusitis, adenoid and tonsil inflammation as well as hypertrophy. Moreover, they provide appropriate environmental conditions for bacteria colonization which in turn leads to allergic reactions and infections. Following the stimulation of infections and allergic reactions, nasal edema, thickening, remodeling, and adenoid and tonsil abnormal hypertrophy occur, leading to multi-planar stenosis of the upper airway. Furthermore, a large amount of secretions during chronic sinusitis can fill the narrow cavity, resulting in the occurrence and development of OSAHS.

Although a large number of studies have demonstrated that adenoid and tonsil hypertrophies are considered the main risk factors of pediatric OSAHS [22, 32], the majority of the studies investigated adenoid size using lateral radiography of the nasopharynx, which excluded the effects of specific embedded type of tonsils on OSAHS. The present study evaluated the tonsil and adenoid sizes of Chinese children using video laryngoscopy and the method of mouth opening and tongue pressing. The study was conducted for the first time in a large sample size. Furthermore, the present study investigated the applications of the aforementioned methods on the evaluation of the severity of OSAHS. Leach et al. retrospectively analyzed in an early study the risk factors of OSAHS. They used the method of mouth opening and tongue pressing, and their data indicated that tonsils of grades III and IV accounted for 76% (26/34) and 40% (18/44) of the subjects in the OSAHS group and in the PSG negative group, respectively. Brooks et al. investigated in 1998 the relationship of the adenoid size with the severity of OSAHS via the measurement of the adenoid size using lateral radiography of the nasopharynx. Their findings revealed that the larger the adenoids, the greater the severity of the OSAHS [33]. In a recent study by Toros et al. it was reported that the larger the tonsils, the greater the severity of the symptoms of OSAHS, such as snoring [34]. In contrast to Toros et al., Tagaya et al. in 2012 suggested that the tonsil size did not significantly correlate with AHI. It was demonstrated that the larger the adenoids, the greater the severity of the OSAHS for preschool children with normal weights [35]. The present study confirmed that the adenoid and tonsil hypertrophies were the main risk factors of pediatric OSAHS, and further demonstrated that adenoid and tonsil sizes positively correlated with the risk and severity of pediatric OSAHS. The risk of pediatric OSAHS with grade IV adenoids was 64 times higher than that with grade III adenoids, while the risk of pediatric OSAHS with grade IV tonsils was 5 times higher than that with grade III tonsils. It may be speculated that grades III to IV of adenoid hypertrophy cause significant stenosis and/or obstruction of the posterior nostrils and nasopharyngeal airway, while grades III to IV of tonsil hypertrophy cause significant stenosis and/or obstruction of the pharyngeal cavity, both of which blocking the respiratory airflow and being the main causes that lead to the development of pediatric OSAHS. Although sufficient space was noted for respiratory airflow in the posterior nostrils, nasopharyngeal airway and pharyngeal cavity, grade I to II adenoids and tonsils can cause OSAHS in association with other factors. The data exhibited a trend of significance with regard to the prevention and treatment of pediatric OSAHS. The treatment methods of OSAHS were different due to the different effects of grades III to IV adenoids and tonsils with regard to the parameters morbidity and disease conditions. Surgical treatment should be used for grade IV adenoids and tonsils, while non-surgical treatment should be applied for grade III adenoids and tonsils. In addition, precautions should be taken for non-OSAHS pediatric patients, since grade IV adenoids and tonsils were demonstrated as high-risk factors for the morbidity of pediatric OSAHS. Nevertheless, the present study used direct visualization techniques to examine the tonsils and novel technologies such as bioimpedance could be examined in this context [36–38].

The determination of the tonsil size should be conducted with caution. Grade I to II tonsils in the case group were detected by the method of mouth opening and tongue pressing. The data indicated that the embedded type of tonsils accounted for 37.1%. A false impression of small tonsils would easily occur upon examination, using the method of mouth opening and tongue pressing. Wong et al. analyzed in 2002 the relationship between the TP value (the ratio of tonsil width to oropharyngeal cavity) and AHI based on the lateral radiography of the nasopharynx. The study concluded that AHI positively correlated with the TP value [39], suggesting the potential effects of the tonsils on the oropharyngeal cavity. Using video laryngoscopy, the larger volume of the embedded type of tonsils and the effects on the oropharyngeal cavity were examined more intuitively. The method of mouth opening and tongue pressing can evaluate the majority of tonsil sizes, although it may lead to misinterpretations with regard to the embedded type of tonsils. Electron-nasopharyngolaryngoscopy can misinterpret the size of the pharyngeal cavity of the tonsil plane. The tonsil size and the space occupied in the pharyngeal cavity can be examined more intuitively, whereas it is helpful to accurately assess tonsil size as well as its effects on pharyngeal stenosis. Moreover, its amplification can clearly display secretions on the surface of tonsils, notably secretions in the upper and lower poles, as well as the medial and dorsal parts that cannot be detected by the method of mouth opening and tongue pressing. Therefore, the method of mouth opening and tongue pressing in combination with video laryngoscopy can offer significant advances in the judgment of the tonsil size and its effects on pharyngeal stenosis. It can further evaluate whether tonsil hypertrophy was developed due to physiological and/or pathological causes and whether the tonsil should be removed by surgical treatment.

Pediatric OSAHS is traditionally attributed to adenoid and tonsil hypertrophies, thus nasal lesions are often dismissed. In 2014, Sin et al. demonstrated that nasal resistance of pediatric patients with OSAHS increased significantly and that it was positively correlated with AHI by comparing the nasal resistance between pediatric patients with OSAHS and normal children [40]. In the present study, the comparison between the case and the control groups confirmed that chronic sinusitis and nasal stenosis were the main risk factors for pediatric OSAHS, which were positively correlated with the severity of OSAHS. Nasal infections and allergic reactions usually induce rhinitis and chronic sinusitis, leading to upper airway stenosis and/or obstruction [41–43]. Since the nasal mucosa of children is delicate and the blood and lymphatic vessels as well as mucous glands are abundant, infections and allergic reactions are more likely to induce rhinitis and chronic sinusitis, thus resulting in nasal stenosis. The data of the present study indicated that all pediatric patients with OSAHS developed varying degrees of rhinitis, chronic sinusitis and/or nasal stenosis, suggesting that the morbidity rate of nasal diseases in pediatric patients with OSAHS was up to 100%. Nasal stenosis caused by rhinitis and chronic sinusitis blocks respiratory airflow, which is a main risk factor for the development of pediatric OSAHS. Chronic sinusitis that does not induce nasal stenosis is also considered a risk factor for pediatric OSAHS, since a large number of secretions are released to the nasal, nasopharyngeal and pharyngeal cavities that lead to severe upper airway obstruction. Rhinitis that does not induce nasal stenosis is not considered a risk factor for pediatric OSAHS, due to a limited number of secretions that are not sufficient to cause upper airway obstruction. Thus, it can be inferred that chronic sinusitis and nasal stenosis are the main causes of pediatric OSAHS, which can co-exist with adenoid and tonsil hypertrophies in order to aggravate OSAHS [44, 45]. Beside infection and inflammation, oxidative stress could also play a role in pediatric OSAHS, since it has been associated with a variety of oral cavity pathologies [46–48], including OSAHS [49, 50]. The use of antioxidants could be used for the control of the abnormal development of adenoid mucosae [51]. Nevertheless, oxidative stress was not assessed in the present study and additional studies are necessary to determine the role of oxidative stress and its control in children with OSAHS.

Since the anterior nares of children (especially infants) are small and the nasal cavity is narrow, the conditions of the nasal cavity are difficult to be clearly displayed by anterior rhinoscopy, which is prone to misdiagnosis. In addition the presence of nasal diseases may be dismissed. Therefore, the diagnosis of pediatric nasal diseases that depends solely on anterior rhinoscopy is not sufficient. Video laryngoscopy and paranasal sinus CT are considered significant methods used for the diagnosis of nasal diseases. In the present study, 260 patients with chronic sinusitis that were diagnosed by video laryngoscopy were enrolled, and 63 (24.3%) out of 260 exhibited no symptoms of chronic sinusitis by CT examination. In the 169 cases of nasal stenosis that were diagnosed by video laryngoscopy, 117 (69.2%) were caused by nasal mucosal swelling and turbinate hypertrophy, while the remaining 52 cases (30.8%) were caused by nasal septum bulging. In the 52 cases of nasal septum bulging, 5 cases (3.0%) presented with nasal septum deviation by CT scan, while the remaining subjects exhibited thickening of the nasal septum mucosa. The results of nasal diseases in pediatric patients with OSAHS by the two examination methods demonstrated that video laryngoscopy was a significant tool for the diagnosis of pediatric chronic sinusitis. Nevertheless, the occurrence of nasal stenosis and the presence of related anatomical abnormalities in the nasal septum required confirmation by CT examination. The majority of the nasal stenosis cases were caused by rhinitis and chronic sinusitis-induced nasal mucosal swelling, thickening and turbinate hypertrophy, while the anatomical abnormalities such as nasal septum deviation were rare.

The contribution of obesity is controversial in the pathogenesis of pediatric OSAHS. In 1992, Leach et al. failed to report an association between obesity and OSAHS [52], but in 2006 Lam et al. revealed a weak correlation between obesity and OSAHS (r = 0.156, *P*< 0.003) [53]. In 2008, Xu et al. demonstrated a strong correlation between obesity and OSAHS (r = 0.535; *P* < 0.001) [22]. In the present study, obesity was not an independent risk factor for non-OSAHS, and there was no significant difference in the grading of the tonsil and adenoid sizes between obesity and non-obesity patients. The PSG examination of the patients in the obesity group indicated a significantly greater severity compared with the non-obesity group, suggesting that obesity may not cause OSAHS directly, although it could be a potential risk factor for a narrow upper airway structure. The fat of obese patients accumulates in the neck, leading to upper airway stenosis and affecting the severity of OSAHS [31, 54–56]. In the present study, 11.5% pediatric patients with OSAHS had craniofacial features such as narrow and long face, micromandible, mandibular retraction, cross-bite and flat bridge of the nose. The majority of pediatric patients (about 82.1%) with the aforementioned craniofacial features experienced moderate and severe OSAHS, although the multivariable analysis indicated that craniofacial deformities were not independent risk factors for non-OSAHS. Thus, it can be concluded that patients with obesity and craniofacial deformities alone do not necessarily develop OSAHS, but they were prone to develop OSAHS in the presence of symptoms such as rhinitis, chronic sinusitis, tonsil hypertrophy and adenoid hypertrophy. Moreover, the disease condition was severe. Therefore, the difference in the severity of the combined diseases for the enrolled patients was one of the causes that led to the controversy with regard to the effects of obesity on the occurrence of OSAHS.

Finally, it should be noted that due to the inconsistency of the diagnostic criteria of the pediatric OSAHS, the following diagnostic criteria were used: AHI higher than and/or equal to 5 times/h (≥5 times/h), as recommended by the Chinese guidelines. Nevertheless, relevant studies have used AHI higher than and/or equal to 1 time/h (≥1 time/h) and/or AHI higher than and/or equal to 1.5 times/h (≥1.5 times/h) as the diagnostic criteria. Consequently, inconsistent diagnostic criteria may be one of the causes leading to differences in the risk factors reported by different studies. In 2005, the study by Valera et al. indicated that the tonsil size correlated with the severity of OSHAS in preschool children, although it had no impact on school-age children [57]. The authors of this study aimed to perform stratification analysis for age and examine the dose-effect relationship of the risk factors that were associated with the severity of OSAHS.

The present study exhibits certain limitations. For example, the evaluation of the sample size was not conducted and this was a single center retrospective case-control study. Consequently, all the data were retrospectively analyzed, indicating potential selection, measurement and recall biases. Moreover, the diagnostic criteria of OSAHS were based on the Chinese guidelines. Thus, the results and the comparison with previous studies should be analyzed with caution.

## Conclusions

In summary, the present study demonstrated that upper respiratory tract infection, allergic reactions-induced adenoid hypertrophy, tonsil hypertrophy, chronic sinusitis, nasal stenosis, certain craniofacial features and obesity may be considered risk factors for the development of pediatric OSAHS. The aforementioned factors correlated with disease severity. Among them, the average number of upper respiratory tract infections each year, adenoid hypertrophy, tonsil hypertrophy, chronic sinusitis and nasal stenosis were independent risk factors for the development of pediatric OSAHS, while craniofacial features and obesity provided a weak association with regard to the upper airway structure in pediatric OSAHS. The determination of the etiology provided the basis for the selection of the treatment methods. Surgery was the main treatment for grade IV adenoids and tonsils, while conservative treatment was the main treatment for grade III adenoids and tonsils. Treatment of rhinitis and chronic sinusitis should be conducted for all pediatric patients regardless of the occurrence of the adenoid and tonsil hypertrophies. The effects of infection, allergic reactions, embedded type of tonsils, craniofacial features and obesity on the occurrence of pediatric OSAHS should be considered during etiology analysis. Taken together, those results suggest that timely treatment of upper respiratory tract infections and control of allergic reactions can reduce tonsil and adenoid hypertrophy and reduce the incidence of OSAHS. Weight loss should also help reduce the incidence of OSAHS.

## Supporting information

S1 Data Raw data. (XLSX)
